# Effects of Arsenic upon the Pulp

**Published:** 1879-09

**Authors:** Louis Ottofy

**Affiliations:** St. Louis, Mo.


### ARTICLE II.

### *The Effects of Arsenic upon the Pulp.*

BY LOUIS OTTOFY, D. D. S., ST. LOUIS, MO.

The facts which will be brought to notice in the course of this paper, are not intended to bring forth new theories or ideas, but only to refresh your minds upon a subject so important. Arsenious acid (as $O_3$,) or the trioxide of arsenic is now almost universally employed in the dental profession, for the destruction of the dental pulp, and is justly claimed to be the best known remedy for that purpose. Before speaking proper of the action of this drug upon the pulp, a few general remarks of its action upon the system at large will not be amiss. Locally applied, it excites violent inflammation and congestion, destroying the vitality of the tissues, and, therefore, can be properly called an escharotic. Internally administered commencing with large doses and continued for a length of time, dryness of the mucous tracts follow, metallic taste, increased flow of saliva, nausea, increased secretion from the liver, bowels and kidneys follow, vomiting of glairy mucous, epigastric pain, irritation of the conjunction iva and soreness of the epigastrium, cutaneous eruptions, loss of the hair and nails, palpitation, oppressed breathing, paralysis, convulsions, conia and delirium, terminating in death. These are briefly stated the successive effects of arsenic upon the system.

The post-mortem indications are deep redness, congestion more properly venous than arterial, erosions, ulceration, softening, effusions of lymph and gangrene of the gastro-intestinal tract. The blood most usually fluid and dark colored, redness of the tracheal, bronchial and pulmonary mucous membrane with venous conjestion of the lungs.

How arsenic does act is a question which has baffled great many investigators. Some claim the poisonous effect to be due to irritation, others to its action upon the nervous and cerebro-spinal system, while some attribute the effect to the severe congestion and strangulation which it causes. That it is not absorbed into the blood has been sufficiently demonstrated; it can be applied to one pulp, and after having destroyed the vitality of that tissue, it can be again applied to other tissues with the same effect, disproving the theory of absorption.

There are two oxides of arsenic, the trioxide (as $O_3$), the one we are in the habit of using, it being superior in action to the other, the entoxide (as $O_5$); both these compounds are available as oxygen carriers; the arsenious acid has no immediate affinity for albumen, but is transformed into a poisonous compound in the system. One peculiarity of arsenic is that the oxides, when in contact with organic substances, are very apt to undergo oxidation and change from one compound into the other and vice versa. If arsenic acid or its weak alkaline salt is digested with fresh albumen, fibrin, pancreas, etc., and even with vegetable protoplasm, dyalisis always yields arsenious acid and no decomposition occurs. Decomposing fibrin gives the same reaction. On the other hand if arsenious acid free or as its salt is digested with pan creas or with the fresh leaves of cabbage, dyalisis yields definite evidence of arsenic acid. Defibrinated arterial blood as well as pure oxyhaemaglobin also undergo this change. It is noticeable in the arsenic eaters, that their respiration is increased in ratio with the amount consumed, therefore, proving that there is an increased oxidation going on, and subsequently an increased amount of carbonic acid eliminated; arsenic, therefore, can not be considered as either promoting constructive or destructive metamorphosis. This all shows that while the arsenic is present in the system, an increased amount of oxidation goes on; which is also the case with arsenious acid itself. When applied to the pulp; it extracts from the blood and tissues oxygen in order to form arsenic acid, and as soon as this is accomplished the tissue is destroyed, in the case of the pulp; at the same time from its tendency to cause inflammation and conjestion an increased amount of blood flows to the pulp, thereby enlarging the caliber of the arterial twigs; the pressure caused by these vessels upon the returning veins causes a stasis of venous blood and strangulation.

Arsenic acid, as mentioned before, if applied to healthy fresh albumen or fibrin yields arsenious acid; the latter on the contrary applied to defibrinated arterial blood as well as pure oxyhaemaglobin, changes to arsenic acid. This, therefore, gentlemen, in conjunction with the strangulating effect of the dilated arteries, tends to prove that death of the pulp takes place from the combined action of strangulation and oxidation.

